# BNT162b2 COVID-19 Vaccine Induced Immune Thrombocytopenic Purpura

**DOI:** 10.1155/2022/5603919

**Published:** 2022-04-12

**Authors:** A. K. Ghosh, S. Bhushan, L. D. R. Lopez, D. Sampat, Z. Salah, C. A. Hatoum

**Affiliations:** ^1^Northeast Georgia Medical Center, Gainesville, GA, USA; ^2^Hematology/Oncology Faculty, Northeast Georgia Medical Center, Gainesville, GA, USA; ^3^Internal Medicine Faculty, Northeast Georgia Medical Center, Gainesville, GA, USA

## Abstract

Immune thrombocytopenic purpura (ITP) has been reported following vaccinations such as MMR as well as after viral infections such as hepatitis C and HIV. Few case reports have been reported of ITP after COVID-19 infections and COVID-19 vaccines. Herein, we present a patient who presented with severe ITP with a platelet count of 0 after receiving the second dose of the BNT162b2 mRNA COVID-19 vaccine (also known as the Pfizer BioNTech). She subsequently recovered with a prolonged treatment course.

## 1. Background

Immune thrombocytopenic purpura (ITP) is a clinical condition where the platelet count is less than 100,000/*μ*L and is associated with petechiae, bruising, or mucosal bleeding when the platelet count is below 50,000/*μ*L [1, 2]. ITP may be primary when autoantibodies to platelets cause platelet destruction without an underlying cause, but it is secondary when it is the result of an associated condition that may include medications, infections, malignancies, or autoimmune conditions. Prior reports have indicated that vaccinations such as MMR have resulted in ITP in children and that viral infections such as hepatitis C, HIV, and EBV infection can be associated with ITP [3–5]. In this report, we present a patient who presented with severe ITP after receiving the second dose of the BNT162b2 mRNA COVID-19 vaccine (also known as the Pfizer BioNTech) and recovered with treatment.

## 2. Case Vignette

A 63-year-old female presented to the emergency department with a rash and easy bruising that started a day after her second dose of BNT162b2 vaccination. The rash was initially noted on both her legs and then spread to the rest of her body. Two days after the second COVID-19 vaccination, the patient noticed a large bruise on her lower back, without prior trauma. In addition, she also noted mild bruising of her tongue but no active bleeding. She reported mild dyspnea after receiving her first COVID-19 vaccine 3 weeks earlier and noted similar symptoms soon after her second dose of the vaccine. Dyspnea was not associated with chest pain or leg edema. The patient denied nausea, vomiting, fevers, chills, cough, sputum production, hematemesis, hematochezia, or any abdominal pain. She had a past medical history of COPD, type 2 diabetes mellitus, and hypertension, but no history of liver disease or bleeding tendencies. She was on hypoglycemic agents, antihypertensives, and citalopram, but not on aspirin or nonsteroidal anti-inflammatory agents.

**Figure 1:**
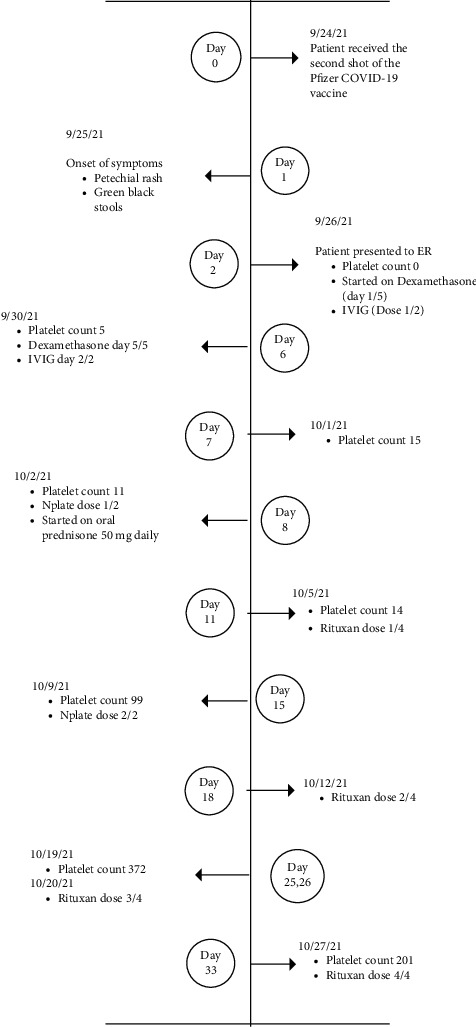
The timeline of the patient's treatments and her platelet counts.

Physical examination revealed generalized petechiae and subcutaneous bruises on the lower back. Laboratory evaluation showed normocytic anemia, normal white blood cell count, and platelet count was 0/*μ*L. Peripheral blood smear was normal except for decreased platelets without evidence of immature platelets.  Figure 1 demonstrates the timeline of the patient's treatments and her platelet counts.

Since thrombocytopenia due to microangiopathic hemolytic anemia (MAHA) can occasionally lead to thromboembolism and, given the patient's initial complaint of dyspnea, with an elevated D-dimer of 0.85, a CT angiogram was ordered. CT angiogram was negative for pulmonary embolism. Subsequent workup including absence of schistocytes on peripheral smear, normal LDH, haptoglobin, and bilirubin levels ruled out MAHA. CT head showed no intracranial bleeding.  Table 1 shows the pertinent lab values at admission and after treatment.

Based on these findings, the patient was diagnosed with immune thrombocytopenic purpura (ITP), believed to be secondary to the COVID-19 vaccine. She was admitted to the hospital. The patient was started on dexamethasone at 40 mg orally daily for 5 days, as well as IVIG 1 g/kg once daily for a total of two doses. Of note, the patient tested positive for Sjogren's Ab (SS-A) and scleroderma antibodies. Workup for other causes of thrombocytopenia including hepatitis serology, HIV, EBV, ANA, Smith Ab, RNP ab, anti-Jo1 Ab, anti-dsDNA Ab, anti-centromere, and Sjogren's ab (SS-B) were negative.

Over the next week, the patient's vitals and overall health remained stable. Her bruising was gradually improving, with no evidence of bleeding or new bruising. While the clinical picture showed stability, her platelet count showed a very slow response to treatment. Labs showed slowly increasing platelet counts from 0K/*μ*L on day 1, 1K/*μ*L on day 3, 5K/*μ*L on day 5, 15K/*μ*L on day 6, 11K/*μ*L on day 7, and 8K/*μ*L on day 8. Since the thrombocytopenia was refractory to IVIG and a 5-day course of steroids, the patient was started on prednisone therapy daily for a total 9 of doses. She subsequently received two doses of Nplate and one dose of Rituxan. Her platelet count responded and improved to 99K/*μ*L on day 14. She was discharged on a prednisone taper and is scheduled to receive the remaining three doses of Rituxan in the outpatient setting.

## 3. Discussion

The diagnosis of ITP is generally made after the exclusion of other causes of thrombocytopenia as well as response to treatment [2]. As in our patient with new onset ITP, the temporal association of the onset of symptoms after the BNT162b2 vaccine is significant but cannot be proved causality. An underlying rheumatological process cannot be entirely ruled out, given patient's mildly positive ANA and positive scleroderma antibodies. A detailed review of the current literature shows that patients have been diagnosed with ITP after COVID-19 vaccination, including some patients with a prior history of ITP and few without prior ITP, as in our patient [6–14]. However, most of the reports have shown ITP occurring after the first dose of vaccine, with few reports after the second dose, as seen in our patient [6, 7, 9–14]. It is also possible that the patient may have had a mild form of ITP after the first dose of the BNT162b2 vaccine but was not symptomatic or not detected at that time.

As most patients with ITP respond well to IVIG and steroids, this is the initial management approach for patients with ITP after the COVID-19 vaccine. If the platelet count remains low, thrombopoietic agents and vincristine may be considered [8]. Rituximab is generally avoided following COVID-19 vaccination due to the slow onset of action, potential negation of recent vaccine-induced immunity, and the inability to revaccinate for more than 6 months [15]. However, it may be administered in refractory cases when the benefits outweigh the risks of treatment [16].

The pathogenesis of ITP is still unclear, but it is believed to be secondary to autoantibodies against platelet membrane glycoprotein by the patient's B cells, or by cytotoxic T cells against platelet precursors [2]. As noted earlier, secondary ITP has been reported following multiple conditions, including vaccinations. Based on a literature review, it may be possible that antibodies directed against an antigen formed by the attachment of vaccine particles to some platelets lead to a reaction involving the rest of the platelets [8]. There have also been reports of ITP following a COVID-19 infection [5, 17]. Interestingly, all the patients presented with symptoms of subcutaneous or mucosal bleeding associated with thrombocytopenia. In general, platelet counts improve rapidly with steroids, but this was not seen in our patient [9]. One speculation regarding the etiology is that an underlying damage to megakaryocytes may have resulted in a lack of platelet production. This could also possibly explain the lack of response to IVIG due to the absence of autoantibodies to platelets. The subsequent improvement after day 14, which included treatment with Nplate, could then have been secondary to the development of a new population of megakaryocytes to reestablish platelet production.

ITP is commonly treated with steroid therapy and IVIG, as in our patient. It is important to highlight that the platelet count responds to therapy with improvement of counts. In some instances, when there are bleeding complications, platelet transfusion may be needed.

In summary, we herein report a unique case of severe ITP likely induced by the Pfizer COVID-19 vaccine, reiterating that this is a rare and treatable condition that can be diagnosed easily and responds to therapy.

## Figures and Tables

**Table 1:** Laboratory values on admission.

Test (reference ranges)	Lab value on admission
*Complete blood cell count*	
Hemoglobin (12.1–15.1 g/dL)	12.7 g/dL
Hematocrit (36.1–44.3%)	37.9%
Platelet counts (150–450 × 10^3^/*μ*l)	0 × 10^3^/*μ*l
White blood cells (4.5–10.0 × 10^3^/*μ*l)	6.8 × 10^3^/*μ*L
Red blood cells (4.2–5.4 × 10^9^/*μ*l)	3.01 × 10^9^/*μ*l

*Coagulation*	
Test (references ranges)	Lab value on admission
Prothrombin time (11–14 seconds)	11.9 seconds
PTT (20–40 seconds)	25.4 seconds
INR (0.9–1.2)	1.04

*Comprehensive metabolic profile*	
Test (references ranges)	Lab value on admission
AST (5–30 IU/L)	33 IU/L
ALT (5–30 IU/L)	44 IU/L
Total bilirubin (0.3–1.9 mg/dL)	0.5 mg/dL
ALP (44–147 IU/L)	39 IU/L
BUN (6–20 mg/dL)	33 mg/dL
Creatinine (0.6–1.2 mg/dL)	1.04 mg/dL
Total protein (6.0–8.3 g/dL)	8.3 g/dL
Albumin (3.5–5.4 g/dL)	2.7 g/dL
Vitamin B12 (130–700 pg/mL)	1469 pg/mL
Folate (2–20 ng/mL)	18.1 ng/ML
Iron (30–170 *μ*g/dL)	75 *μ*g/dL
LDH (50–150 IU/L)	131 IU/L
Haptoglobin (41–165 mg/dL)	177.0 mg/dL
D-dimer (<0.5 *μ*g/mL)	0.850 *μ*g/mL
